# Clinicopathologic Analysis of Granuloma Annulare With Insights Into Its Incidence and Demographics in the United Arab Emirates From 2016 to 2023

**DOI:** 10.7759/cureus.84287

**Published:** 2025-05-17

**Authors:** Farah Awadhalla, Hamda Alfalasi, Reem Elbahtimi

**Affiliations:** 1 Family Medicine, Mohammed Bin Rashid University of Medicine and Health Sciences, Dubai, ARE; 2 Dermatology, American Hospital Dubai, Dubai, ARE; 3 Dermatopathology, International Dermpath Consult, Dubai, ARE

**Keywords:** clinicopathology, dermatology, epidemiology, granuloma annulare, uae

## Abstract

Granuloma annulare (GA) is a benign, often chronic dermatologic condition with varying clinical presentations and has been associated in the literature with a notable psychosocial burden, particularly in generalized cases. This retrospective cross-sectional study analyzed the epidemiological and clinicopathological features of GA and its variants in the United Arab Emirates (UAE) from 2016 to 2023. A total of 171 histopathologically confirmed GA cases were included, collected from a single dermatopathology center. The incidence of GA diagnoses showed temporal variation, peaking in 2020 at 0.67% (n = 27) and declining significantly to 0.27% (n = 20) by 2023, as confirmed by a two-proportion z-test (z = 3.24, p = 0.001). However, no significant monotonic trend was observed across the full eight-year period (ρ = -0.19, p = 0.65). Localized GA emerged as the predominant subtype (n = 118, 69.0%), with a marked female predominance across the cohort. The mean age of patients was 39.49 ± 13.87 years, and middle-aged adults (36-55 years) constituted the largest age group (n = 82, 48%). A chi-square goodness-of-fit test demonstrated a statistically significant difference in the distribution of GA variants (χ²(3, N = 171) = 186.54, p < 0.001), with interstitial, papular, and deep GA occurring less frequently than expected. Lesions most frequently involved the upper and lower limbs. While age distribution across GA subtypes did not show a statistically significant association (χ²(12, N = 171) = 13.62, p = 0.326), demographic patterns revealed a richly diverse patient population representing over 35 nationalities, with British and Emirati individuals comprising a notable portion. These findings contribute meaningful insight into the epidemiology and clinicopathology of GA in a multicultural setting and offer a foundation for further exploration into immunologic and genetic factors that could drive precision-based treatment strategies.

## Introduction

The United Arab Emirates (UAE) is a nation distinguished by its cultural richness and demographic diversity. A defining cultural aspect is the prevalence of consanguinity, with rates ranging from 40% to 60% [1]. This cultural practice has significantly contributed to the increased incidence of genetic disorders within the Emirati population. A comprehensive review identified 665 distinct genetic conditions among Emiratis, with over half being extremely rare and predominantly inherited in an autosomal recessive manner [2].

Granuloma annulare (GA) is a relatively common dermatological condition characterized by granulomatous inflammation in the skin. Typically presenting as erythematous plaques or papules arranged in an annular configuration, GA is often benign and self-limiting, though its etiology remains poorly understood. Pathologically, GA is marked by focal collagen degeneration, mucin deposition, and an infiltrate of histiocytes and lymphocytes, manifesting in distinct patterns such as interstitial, palisaded, sarcoidal, and mixed granuloma [3]. The most frequent presentation is the interstitial pattern, where histiocytes are scattered among collagen bundles in the dermis, while the second most common is the palisaded granulomatous pattern, which is characterized by a ring of histiocytes encircling necrobiotic collagen [4]. The localized form predominantly affects the distal extremities, particularly the hands and feet [5]. Generalized GA can involve a broader distribution, including the trunk and limbs [6]. Subcutaneous GA is more common in children and typically presents as asymptomatic, firm nodules on the lower legs, hands, scalp, and buttocks [7].

GA has been studied extensively for its potential associations with systemic diseases. Although most cases are idiopathic, sporadic links with autoimmune diseases, particularly diabetes mellitus, thyroid disorders, and infectious agents such as HIV, have been noted [8]. These associations underscore the importance of understanding the epidemiology of GA, particularly in clinical settings where these conditions may overlap. Additionally, distinguishing GA from other granulomatous conditions, such as sarcoidosis, is critical due to the prognostic implications, as sarcoidosis can involve systemic organs [9].

Demographic patterns of GA have shown slight variations across populations. Most studies indicate a female predominance, with a peak incidence in the first three decades of life [10]. Localized GA, the most common variant, primarily affects the extremities, particularly the dorsal hands and feet. Other variants, such as generalized GA, occur less frequently but may present with more extensive skin involvement, raising concern for systemic disease [11]. Despite its benign nature, previous studies have reported that patients with extensive or recurrent lesions may experience significant psychological distress, highlighting the importance of understanding the factors contributing to disease persistence [12].

Epidemiological studies of GA in non-Western populations are limited. In the UAE, which has a diverse population of both nationals and expatriates, this study provides a unique opportunity to examine GA across different ethnic and socioeconomic groups. Previous studies, primarily from Europe [13] and North America [14], have largely focused on Caucasian populations, with little data available from the Middle East. This gap in the literature underscores the importance of studying GA in the UAE, where environmental, genetic, and lifestyle factors may differ significantly from Western settings.

The current study aims to address this gap by investigating the clinicopathologic characteristics and demographic patterns of GA in the UAE from 2016 to 2023. Utilizing data from International Dermpath Consult (a private laboratory for skin pathology), this retrospective observational study includes patients from various ethnic backgrounds. By analyzing the relationship between GA variants and demographic factors such as age, gender, and nationality, the study seeks to provide a comprehensive understanding of GA within this diverse population.

Recent advances in dermatopathology have provided new insights into the pathophysiology of granulomatous diseases such as GA. Studies have identified key immune pathways involved in granuloma formation, particularly involving T-helper cells, tumor necrosis factor-alpha (TNF-α), and interleukin-1 (IL-1) [15]. These cytokines play a central role in the activation of macrophages and the formation of multinucleated giant cells. In GA, these inflammatory responses are typically well-contained and non-destructive, in contrast to more aggressive granulomatous diseases such as sarcoidosis [16].

Genetic predisposition may also contribute to the development of GA. Although specific genetic markers have yet to be identified, there is evidence to suggest familial clustering in some cases, indicating a potential hereditary component [17,18]. Further research into genetic susceptibility, particularly in populations with high consanguinity such as in the Middle East, could provide valuable insights into the etiology of GA [19].

Managing GA, particularly in generalized or treatment-resistant cases, remains challenging due to the inconsistent efficacy of available therapies. For localized GA, conservative approaches are often recommended, with spontaneous resolution occurring within months to years. Topical corticosteroids can provide symptomatic relief, but their long-term efficacy is variable [7,20]. In more severe cases, systemic treatments such as hydroxychloroquine and dapsone are often used, although relapses are common after discontinuation [21]. Emerging therapies, including TNF-α inhibitors, show promise but require further research to confirm their safety and effectiveness [22]. Identifying genetic and environmental risk factors, especially in populations like the UAE where consanguinity may play a role, could help guide treatment decisions and improve patient outcomes.

Despite therapeutic advancements, a significant proportion of GA cases remain chronic and require ongoing clinical attention. While prior studies have explored various clinical subtypes and their potential psychosocial implications [23], there remains a need for region-specific data to better inform diagnosis and management strategies. This study aims to characterize the epidemiological and clinicopathological features of GA and its variants in the UAE, providing insights into the subtype distribution, demographic patterns, and trends across a diverse population.

## Materials and methods

Study design

This retrospective observational cross-sectional study was conducted to evaluate the epidemiological characteristics of GA and its variants in the UAE from 2016 to 2023. The study adhered to the Strengthening the Reporting of Observational Studies in Epidemiology (STROBE) guidelines to ensure comprehensive and transparent reporting.

Study population

The study included all patients diagnosed with GA between 2016 and 2023 at International Dermpath Consult. Inclusion criteria comprised individuals with a confirmed histopathological diagnosis of GA, including its variants (localized, generalized, interstitial, and palisaded GA). GA subtypes were classified based on histopathological and clinical criteria. Localized GA was defined as annular or arcuate plaques limited to one or two body sites. Generalized GA was characterized by widespread lesions involving multiple body regions. Interstitial GA demonstrated a histologic pattern of interstitial histiocytes, and lymphocytes dispersed between collagen bundles, without palisading granulomas. Papular GA was identified by small, grouped papules without annular configuration. Deep GA was diagnosed based on the presence of granulomatous inflammation extending into the deep dermis or subcutaneous tissue, typically forming nodules. All cases were confirmed via histopathology by a dermatopathologist. Patients diagnosed with other granulomatous conditions, such as sarcoidosis or tuberculosis, were excluded to maintain diagnostic specificity.

Data collection

Data were retrospectively extracted from the pathology database provided by Dr. Reem El Batimi. Collected variables included patient age, gender, nationality (classified as UAE national or expatriate), and the anatomical site of the lesion. Age at diagnosis was recorded in 10-year intervals to allow for analysis of age-specific incidence rates. The histopathological subtype of GA was categorized into relevant variants, including interstitial and palisaded types.

Study size

The total study size (n = 171) was determined by the number of patients meeting the inclusion criteria, consisting of all cases diagnosed as GA within the specified timeframe.

Bias and limitations

Potential selection bias was acknowledged, as the study relied on cases from a single dermatopathology center, limiting generalizability. Additionally, not all nationalities were consistently documented, which could affect the demographic analysis.

Statistical methods

All statistical analyses were conducted to evaluate trends, associations, and distributional differences within the study population. The incidence rates of GA diagnoses from 2016 to 2023 were analyzed using Spearman’s rank-order correlation to assess monotonic trends over time. A two-proportion z-test was performed to compare the incidence rate in 2020 (the peak year) with that in 2023 to determine whether the observed decrease was statistically significant. To examine the distribution of GA variants and site of lesions, a chi-square goodness-of-fit test was used to assess whether the observed frequencies of variant types and lesion sites differed significantly from expected proportions. Additionally, descriptive statistics (mean, standard deviation, and range) were used to summarize the age distribution across GA variant groups. a chi-square test of independence was conducted to explore potential associations between categorical age groups and GA subtypes. A p-value of <0.05 was considered statistically significant for all inferential tests. Analyses were performed using IBM SPSS Statistics Version 28.0 (IBM Corp., Armonk, NY) and RStudio.

## Results

Incidence rate

**Figure 1:**
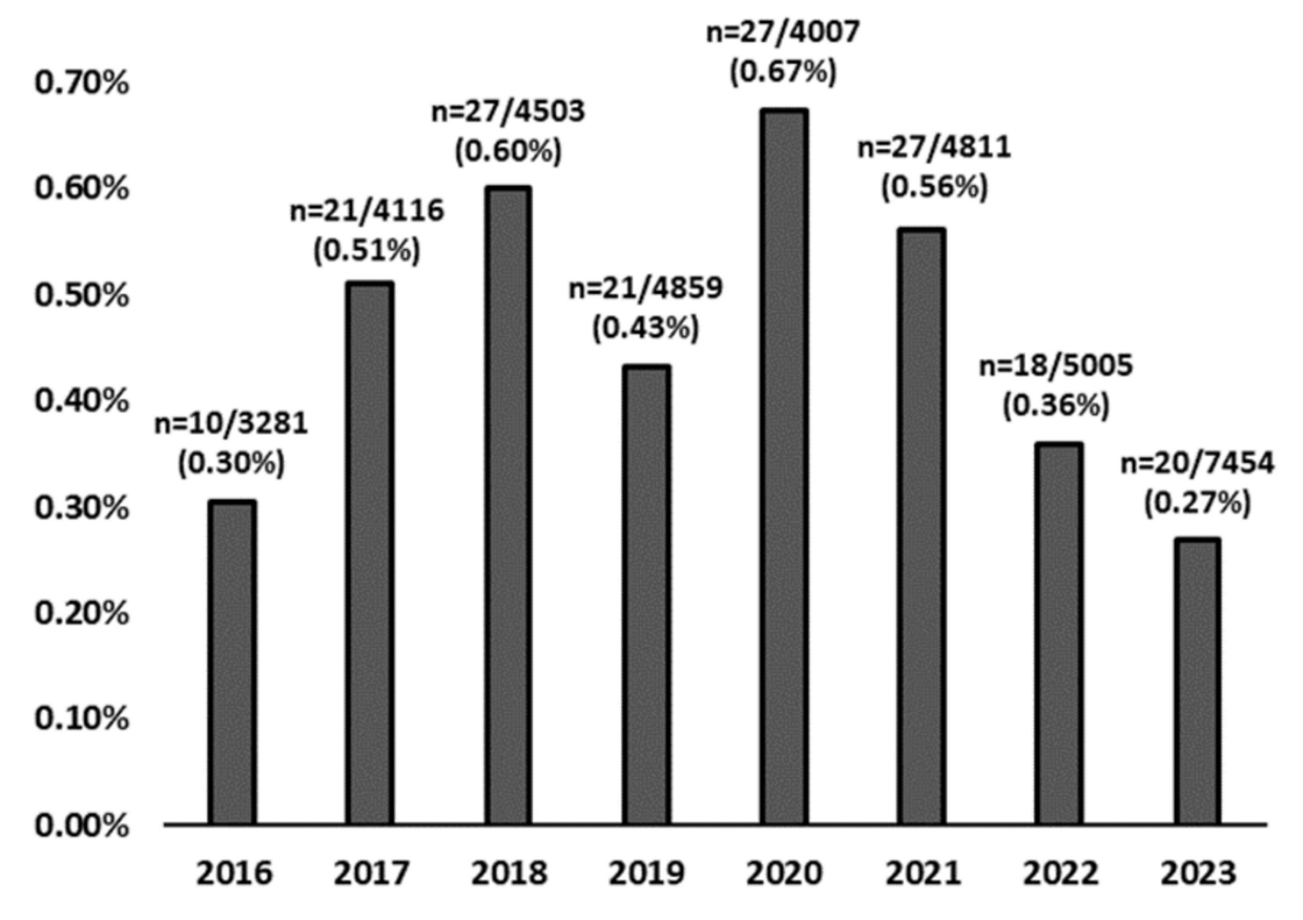
Incidence rates of GA diagnoses from 2016 to 2023. Spearman’s correlation showed no significant trend over time (ρ = –0.19, p = 0.65); however, incidence dropped significantly from 2020 to 2023 (z = 3.24, p = 0.001). GA, granuloma annulare

The incidence rates of GA diagnoses from 2016 to 2023 (Figure 1) were analyzed to assess temporal trends and notable changes over time. The percentage in Figure 1 reflects the proportional frequency of GA cases among all skin biopsies processed at the dermatopathology center each year. Spearman's rank-order correlation revealed no significant monotonic trend in incidence rates across the eight-year period, ρ(6) = - 0.19, p = 0.65. However, a two-proportion z-test comparing the peak incidence in 2020 (0.67%) to the rate in 2023 (0.27%) revealed a statistically significant decrease, z = 3.24, p = 0.001. These results suggest that while the overall incidence rates did not follow a consistent upward or downward trajectory, the observed decline from 2020 to 2023 represents a meaningful and statistically significant reduction.

Gender distribution

**Figure 2:**
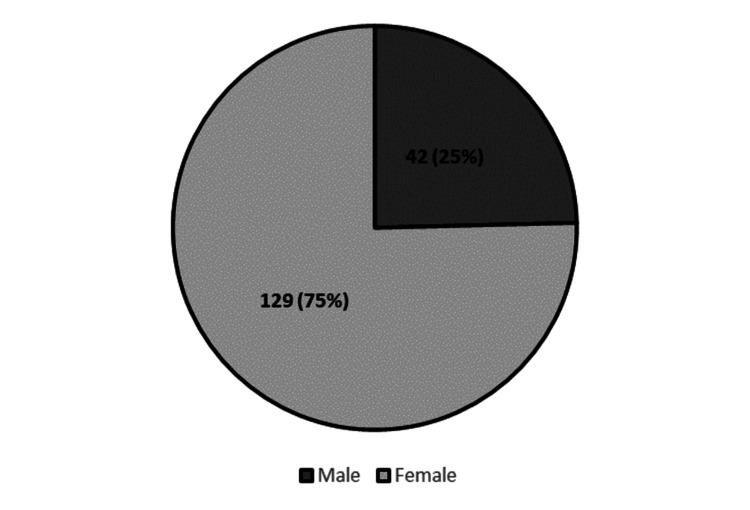
Gender distribution of the study population (N= 171).

A comparison of incidence rates by gender indicates that the majority of diagnoses were among females (Figure 2).

GA variants

**Figure 3:**
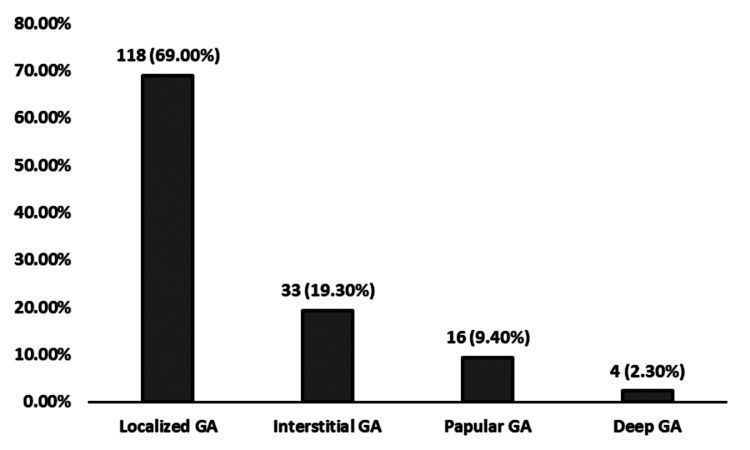
Distribution of GA variants in the study population (N= 171). A chi-square goodness-of-fit test revealed a significant difference in the distribution of GA variants, χ²(3, N = 171) = 186.54, p < 0.001. GA, granuloma annulare

A chi-square goodness-of-fit test revealed a significant difference in the distribution of GA variants, χ²(3, N = 171) = 186.54, p < 0.001. Localized GA was observed significantly more frequently (n = 118), while interstitial GA (n = 33), papular GA (n = 16), and deep GA (n = 4) occurred less often than expected (Figure 3).

Lesion distribution across body regions

**Figure 4:**
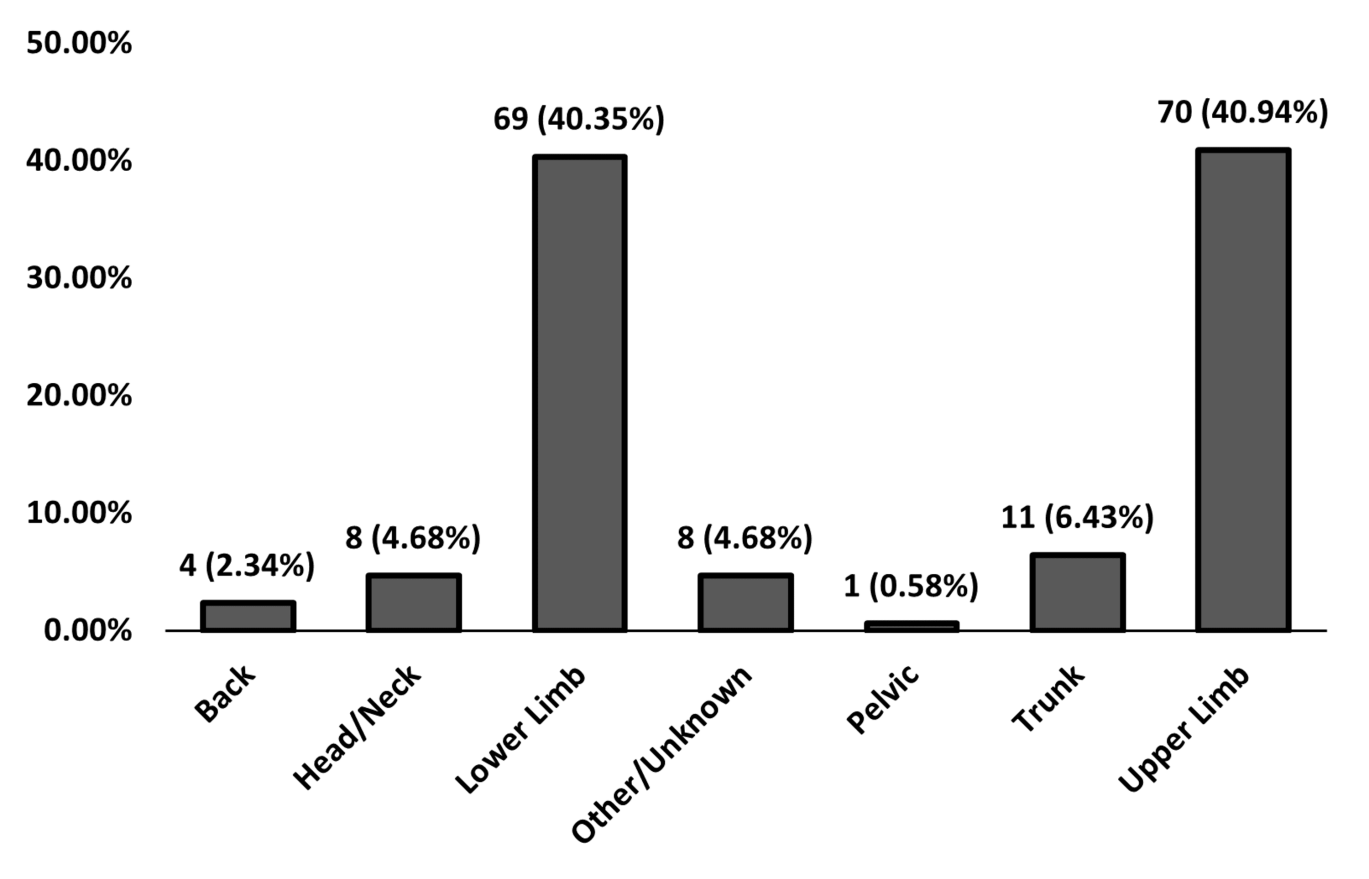
Distribution of lesion sites across body regions in the study population (N = 171). A chi-square goodness-of-fit test indicated a significant difference in the distribution of lesion sites, χ²(6, N = 171) = 235.37, p < 0.001.

A chi-square goodness-of-fit test showed a significant difference in lesion distribution across body regions, χ²(6) = 235.37, p < 0.001. Lesions were most common in the lower limb (n = 69) and Upper Limb (n = 70), while the pelvic region had the fewest reported cases (n = 1) (Figure 4).

Age distribution across different GA variants

The descriptive statistics for age indicate that the sample consists of 171 patients, with ages ranging from 2 to 75 years. The mean age of the patients was 39.49± 13.87 years, suggesting a relatively diverse age distribution within the sample (Table 1).

**Table 1:** Age statistics by GA variant GA, granuloma annulare

GA Variant	Age Statistics (Mean ± SD, Min-Max)
Localized GA	39.62 ± 14.27 (2.0-75.0)
Interstitial GA	40.55 ± 13.61 (7.0-70.0)
Papular GA	38.06 ± 12.47 (17.0-60.0)
Deep GA	32.75 ± 10.87 (26.0-49.0)

Relationship between age categories and diagnosis types

Age was categorized into five groups: children (2-12 years), teenagers (13-19 years), young adults (20-35 years), middle-aged adults (36-55 years), and older adults (56-75 years), as shown in Table 2. Among 171 cases, middle-aged adults were the largest group (n = 82), with localized diagnoses being the most common (n = 53). A chi-square analysis showed no significant link between age and diagnosis types, χ²(12, N = 171) = 13.62, p = 0.326.

**Table 2:** Distribution of GA variants across age categories (count and percentage) A chi-square test of independence showed a non-significant difference among different age categories: χ²(12, N = 171) = 13.62, p = 0.326. GA, granuloma annulare

Age Category	Localized GA	Interstitial GA	Papular GA	Deep GA
Children (2-12 years)	7 (77.80%)	2 (22.20%)	0 (0.00%)	0 (0.00%)
Teenagers (13-19 years)	1 (50.00%)	0 (0.00%)	1 (50.00%)	0 (0.00%)
Young adults (20-35 years)	42 (72.40%)	6 (10.30%)	7 (12.10%)	3 (5.20%)
Middle-aged adults (36-55 years)	53 (64.60%)	21 (25.60%)	7 (8.50%)	1 (1.20%)
Old adults (56 to 75 years)	15 (75.00%)	4 (20.00%)	1 (5.00%)	0 (0.00%)

Nationality

The nationality distribution of the participants in this study is shown in Table 3.

**Table 3:** Frequency distribution of nationalities in the study of granuloma annulare from 2016 to 2023

Nationality	Frequency	Percent
Afghan	1	0.6
Algerian	1	0.6
American	7	4.1
Australian	3	1.8
Bangladeshi	1	0.6
Belgian	1	0.6
Brazilian	2	1.2
British	20	11.7
Bulgarian	2	1.2
Canadian	1	0.6
Czech	1	0.6
Danish	1	0.6
Egyptian	3	1.8
Emirati	11	6.4
Filipino	3	1.8
French	4	2.3
German	1	0.6
Indian	10	5.8
Iranian	1	0.6
Iraqi	1	0.6
Irish	1	0.6
Japanese	1	0.6
Jordanian	7	4.1
Lebanese	7	4.1
Mexican	2	1.2
Netherlander	1	0.6
Norwegian	1	0.6
Pakistani	3	1.8
Palestine	2	1.2
Portuguese	1	0.6
Romanian	1	0.6
Russian	1	0.6
Sierra Leonean	1	0.6
Spaniard	2	1.2
Sudanese	1	0.6
Syrian	5	2.9
Turk, Turkish	1	0.6
Ukrainian	2	1.2
Total	171	100

## Discussion

This retrospective observational study provides a comprehensive analysis of GA in the UAE from 2016 to 2023. The overall incidence of GA diagnoses in the UAE demonstrated a gradual increase, peaking in 2020 with a notable decline in subsequent years. This trend could be attributed to various factors, including environmental changes, increased awareness of the condition, or shifts in healthcare accessibility during the COVID-19 pandemic. The fluctuation in incidence rates aligns with other studies that have observed temporal variability in dermatological conditions, often influenced by seasonal changes, public health interventions, and infectious outbreaks [24].

The study also confirms previous findings that GA predominantly affects females, with a female-to-male ratio consistent with global data [14,25]. This gender predominance might be linked to hormonal influences or genetic predispositions that require further investigation [26]. In the UAE, cultural and lifestyle factors, such as differences in healthcare-seeking behavior between men and women, may also contribute to the higher diagnosis rates among females [27]. Studies have also shown that GA can exhibit worsening in sun-exposed areas in certain cases, indicating a possible photosensitive response. Interestingly, while some patients report aggravation with UV exposure, others have demonstrated improvement during summertime or with phototherapy. This dual response highlights the complex interplay between ultraviolet light and GA [28].

The significant predominance of localized GA (69%) in this study aligns with the higher rates of localized GA reported in Western populations [29]. The observed predominance of localized GA in this study is consistent with recent findings. Joshi and Duvic reported that localized GA accounts for approximately 75% of cases [7].

Histopathologically, interstitial GA was the second most common subtype, reflecting global trends where interstitial patterns are often the most frequent histological presentation [3]. However, the rarity of papular GA in this study (9.4%) may highlight regional differences in the manifestation of granulomatous diseases [30]. The identification of histopathological variants is essential for distinguishing GA from other granulomatous conditions such as sarcoidosis, which may require more aggressive management due to potential systemic involvement [31]. The age distributions for GA variants observed in this study align with existing literature, with localized GA common in younger adults, generalized forms including interstitial and papular types in older individuals, and deep GA (subcutaneous) primarily in children [6].

One of the unique contributions of this study is its focus on the nationality of GA patients, which reflects the diverse ethnic composition of the UAE. British nationals constituted the largest proportion of the study sample (11.7%), followed by Emirati nationals (6.4%) and Indian nationals (5.8%). The relatively high number of British patients may be indicative of the expatriate population demographics in the UAE. Furthermore, the fact that Emirati nationals represented a significant portion of the cohort suggests that GA may be more prevalent in this population than previously recognized, although the exact incidence rates for UAE nationals compared to expatriates are difficult to assess without larger population studies.

In comparison to studies conducted in Europe and North America, where Caucasian populations dominate the study samples [7,20,32], the inclusion of a more ethnically diverse cohort in the UAE provides valuable insight into how GA affects different ethnic groups. The potential role of genetic factors, especially in populations with higher rates of consanguinity, as seen in some Middle Eastern communities, should be explored further [33]. Previous research has indicated familial clustering of GA, suggesting a hereditary component that may be particularly relevant in populations with a high incidence of consanguineous marriages [34,35].

Managing GA, particularly in patients with generalized or recurrent disease, remains challenging. The chronic nature of the condition has been associated in prior studies with psychological distress, emphasizing the importance of comprehensive care strategies. Although localized GA often resolves spontaneously, generalized GA carries a higher risk of recurrence and has been linked to greater emotional burden [7]. While our study did not directly assess psychosocial outcomes, it contributes to understanding the clinical burden of generalized GA in the UAE. In this context, it is also important to consider that psychological support for affected individuals may be underutilized due to cultural stigmas surrounding mental health.

In terms of treatment, systemic therapies such as hydroxychloroquine and dapsone have shown variable success in managing generalized GA [7,36]. Emerging treatments, such as TNF-α inhibitors, have shown promise in severe or treatment-resistant cases [37]. However, given the limited availability of these therapies in certain regions, including the Middle East, and the high cost associated with them, there is a need for more research on accessible and cost-effective treatment options tailored to the regional healthcare infrastructure.

While this study offers valuable insight into the clinicopathologic patterns of GA in a diverse UAE population, certain limitations should be noted. The single-center design and retrospective data collection may limit generalizability. Nationality data were not consistently available for all patients, and comorbidity information such as diabetes could not be assessed. Subtype classification was based on histopathological features, though definitions were applied retrospectively. Nonetheless, these findings provide a strong foundation for future multi-center, prospective studies to further explore GA’s clinical diversity and contributing factors.

## Conclusions

This study provides valuable insights into GA in the UAE, with the highest prevalence observed in localized GA, particularly among females, reflecting global patterns. The findings underscore the need for further research into gender-specific and genetic factors influencing GA. The UAE’s unique demographics and high consanguinity rates offer critical perspectives on disease etiology. By focusing on a non-Western population, this research contributes to the broader understanding of GA and highlights the importance of localized studies for effective clinical management.
